# Prevalence and correlates of sexual violence among adolescent girls and young women: findings from a cross-sectional study in a South African university

**DOI:** 10.1186/s12905-021-01445-8

**Published:** 2021-08-16

**Authors:** Anthony Idowu Ajayi, Elmon Mudefi, Eyitayo Omolara Owolabi

**Affiliations:** 1 Population Dynamics and Sexual and Reproductive Health, African Population and Health Research Centre, APHRC Campus, Off Kirawa Road, Manga Close, Kenya; 2 Department of Sociology, University of Fort Hare, 50 Church Street, East London, South Africa; 3 Centre for Global Surgery, Department of Global Health, Stellenbosch University, Cape Town, South Africa

**Keywords:** Sexual violence, Alcohol use, Family financial support, Rape, South Africa

## Abstract

**Background:**

Epidemiological data on the prevalence and factors associated with sexual violence is critical to understanding the magnitude of the problem and designing effective interventions. Drawing from cross-sectional data from a South African university, we examined the prevalence and correlates of sexual violence among adolescent girls and young women (AGYW).

**Methods:**

We analysed data of 451 AGYW selected using stratified sampling. Sexual violence was defined as any sexual acts, and attempt to obtain sexual acts without consent. We used adjusted and unadjusted logistic regression models to examine the factors associated with exposure to sexual violence while controlling for relevant covariates.

**Results:**

The lifetime and past-year prevalence of sexual violence was 37.9% and 25.3%, respectively. A higher prevalence of sexual violence was reported by heavy episodic drinkers of alcohol (lifetime 48.4% and past year 34.0%), those who received insufficient financial support (lifetime 58.0% and past year 35.8%) compared to non-users of alcohol (lifetime 27.2% and past year 17.2%),) and those who received adequate financial support (lifetime 20.8% and past year 13.1%). AGYW who reported heavy episodic use of alcohol had higher odds of reporting lifetime (AOR: 1.86; 95% CI: 1.07–3.25) and past year (AOR: 2.03; 95% CI: 1.10–3.75) experience of sexual violence compared to non-users. However, individuals who received adequate family financial support were 76% and 65% less likely to report lifetime and past year experience of sexual violence than those who received inadequate family financial support. Also, AGYW who rated themselves as very religious were 80% and 75% less likely to report lifetime and past year experience of sexual violence compared to those who were not religious.

**Conclusion:**

Our study shows that sexual violence affects a large proportion of girls, requiring intervention that not only focuses on increasing social support for survivors, facilitating reporting, and ensuring perpetrators are convicted, but also target alcohol use reduction and poverty alleviation.

## Background

Sexual violence is a significant and pervasive public health concern, yet scarcely reported and investigated by appropriate authorities, especially in sub-Saharan Africa [1, 2]. It has several devastating mental, physical, social and somatic effects such as poor relationships with peers and increased psychological disorders like depression, anxiety, insomnia, posttraumatic stress disorder, self-harm, poor academic performance, and physical disability [3–6]. Other adverse consequences of sexual violence include an increased likelihood of engaging in risky sexual behaviours and alcohol abuse as well as an increased risk for sexually transmitted infections (STIs), including HIV/AIDS [7–9].

The majority of cases of sexual violence takes place before age 24, and the first year of study is understood to be the most vulnerable year for sexual violence occurrence [4, 10], thus, making college campuses a high-risk environment and a significant and strategic place for interventions aimed at combating sexual violence [11, 12]. Women are particularly at a higher risk, and at least one in five women experience sexual violence or assault during their college years [1, 13]. Various individual-level factors such as multiple sexual partners[14], alcohol or substance abuse [4], prior history of victimisation [4, 15], and socio-economic status [16, 17] further contribute to the increasing burden of sexual violence. Also, institutional-level factors such as lack of effective policies and support for victims [13] foster the growing burden of sexual violence. Likewise, some traditions, such as patriarchal practices, portray women as subordinates, promote sexual violence, and predispose women to higher risk [18, 19].

Although there seems to be an increasing sensitisation to this pressing problem across various countries, there is still a low rate of reporting. The weak justice system, shame, fear of not being believed, and social norms such as the "rape myth" are some of the factors that hinder proper reporting of incidences by victims [20, 21]. Choo and Dunne[22] also implicated a low level of awareness of the various forms of sexual violence activities, especially the non-penetrative acts as another factor contributing to the low reportage of the incidence among the victims. Worryingly, the burden of sexual violence and the associated impacts are not well understood due to poor documentation and under-reporting, which affects the development of effective measures and policies for combating the challenge [23, 24]. Unless the silence around sexual violence incidence is broken, the burden might linger for long [25].

South Africa is faced with an exceedingly high rate of sexual violence, with studies reporting a lifetime prevalence of 26.2% in academic institutions [26] and 24.9% among women aged 18–49 years[27]. These prevalence are higher than the global prevalence of 7.2% [28] and 17.4% in Southern Africa [28]. The judicial structures in the country have failed to convict offenders in many cases of sexual violence, which has, to some extent, worsen the problem [29], resulting in a low level of reporting [30]. Victims are often scared of the justice system and the possibility of re-traumatisation [30]. Naidoo and Moffat [30] further reiterated that only one out of nine cases of sexual violence gets reported. There is a resultant promotion of a "rape culture," which leads to acceptance of this act. Even though many cases of rape and murder of young girls have received large media coverage, the recent rape and killing of a young university girl in Cape Town shook the conscience of the nation, with the president personally attending her funeral. In response to this incidence, the South African government has organised a ministerial task committee for creating a support system for victims and ensuring justice is established. We are, however, unsure of how far-reaching this effort would be.

Also, only a few studies have reported epidemiology data on sexual violence among young people in the country. A nationally representative study of sexual violence among children in South Africa reported a lifetime prevalence of 10% and 15% among boys and girls, respectively[31]. The study further claims that physical abuse, emotional abuse, neglect, family violence and substance misuse were strongly associated with sexual violence. Another study shows that sexual violence clusters around both primary and secondary schools in low-income settings in South Africa[32]. Overall, epidemiology data on prevalence and correlates of sexual violence among young people, especially university students, are lacking in South Africa. Academic institutions across the country are in the process of enacting and implementing policies centred around awareness creation on sexual violence, ensuring justice for victims, providing support for victims, and ultimately reducing the burden of sexual violence [25], the majority of which lacks relevant framework and are poorly implemented [33]. These efforts are better implemented with adequate data and the identification of contributing or associated factors. Reliable epidemiologic data is required to formulate evidence-based and effective policies to combat sexual violence at both the institution and the population level [7, 34]. Therefore, this study aimed to determine the prevalence and associated factors of sexual violence among adolescent girls and young women (AGYW) in one of the economically disadvantaged universities in South Africa. The study's findings could help ascertain the magnitude of the burden, which in turn could inform better policy formulation and implementation of intervention strategies.

## Methods

### Study Context and design

We analysed data from a larger study that examined the individual and family-related factors that threaten the sexual health of adolescents and young adults (aged 17–24 years) [35, 36]. This analysis is motivated by the recent national attention focusing on addressing sexual violence following the shocking rape and murder of a first-year student of the University of Cape Town. The study was conducted at a university campus in the Eastern Cape Province of South Africa between June and November 2018. The campus has a population of 6,000 students.

### Sampling and study procedure

A total of 833 male and female students took part in the main study. The study sample size of 833 respondents was estimated using the MaCorr Sample Size Calculator, at ± 3.3% precision level, a 95% confidence level, and a population of 6,000 students. To ensure representativeness, participants were selected using stratified sampling. Stratification was based on sex, year and faculty of study. The parent study included more females than males, given that female students accounted for over 60% of the students' population on campus. The samples selected in each year and faculty of study were proportionate to the size of these strata. For students to be eligible for this study, they have to be unmarried and aged 24 years or below and mentally capable of responding to the study questions. Eligible participants were recruited in their lecture room by trained research assistants and were asked to consent to the study after they have understood the purpose of the study and measures to protect their privacy, anonymity, and confidentiality. Questionnaires were self-administered in a private space earmarked for the study on campus and via the ODK application for android. Overall, 477 females completed the questionnaire. However, this analysis was limited to only 451 female participants (86.6% response rate) with complete responses to the question on sexual violence.

A sample of 451 is sufficient to yield 80% statistical power, at a ± 4.6 precision, 95% confidence level and 50% maximum variation. The parent study collected self-reported information on HIV testing, knowledge and use of pre and post-exposure prophylaxes, condom use self-efficacy, sexual behaviours, contraceptive use, unintended pregnancy, abortion, contraceptive satisfaction, and sexual violence. Research assistants were explicitly trained for this study on how to use smartphones with an open data kit (ODK) application to administer the questionnaire. A pilot survey with 30 students from nearby Walter Sisulu University was first carried out to validate and further improve the questionnaire. Before administering each questionnaire, research participants were first furnished with information about the purpose of the study, the process involved, and the use of findings. The University of Fort Hare ethical review body granted ethical clearance. Only respondents who voluntarily accepted to participate in this study were interviewed after signing an informed consent form. Furthermore, researchers upheld all ethical research considerations that include confidentiality of provided information and anonymity of participants. Lastly, this study followed all the prescribed IRB guidelines for research when using human subjects.

### Measures

#### Dependent variable

The dependent variable for this study was sexual violence. We defined sexual violence as being forced to perform any sexual act, including being physically forced to have sexual intercourse or unwanted touching of genitals. Participants were asked whether or not they had ever experienced any form of sexual violence and if they did in the past year. Sexual violence was described to them in the questionnaire as unwanted and inappropriate touching of genitalia, coerced sex and rape. Participants were required to select the dichotomously provided responses of either a 'Yes' or a 'No'. We also asked who the perpetrator was and whether they informed anyone about the incident.

#### Independent variables

The independent variables included are demographic characteristics, such as age and parity, family factors such as family structure, living with parents and family financial support, and behavioural factors such as smoking tobacco products and alcohol and drug use. The selected variables are based on our review of the previous studies [4, 16, 17]. Age was measured as a continuous variable but later categorised as 19 years or less and 20–24 years. Parity was assessed by asking respondents how many children they have. We asked participants to select their family type from a list containing both parent family, single-parent family, and foster family. Also, participants were asked to rate the level of financial support they received from their family as "no support," "insufficient support," "moderate support," and adequate support. Family financial support in this study is a subjective measure of family wealth since AGYW may not accurately report their parental income. By making them report on how they feel about the totality of the financial support they get from their parents and guardians, we are able to assess their socio-economic vulnerability. We further asked respondents if their mother and father are alive and whether they live with them. Alcohol use was categorised based on their ‘ever used’, current use and 'never used' of alcohol. In addition, we asked if respondents had ever smoked, currently smoke, and never smoked any tobacco products. Lastly, we asked the participants if they have ever and currently use drugs like cannabis, dagga, cocaine, and tik for recreational purposes. To ascertain drinking intensity among those who drink, we asked respondents to indicate the highest number of standard alcoholic drinks they have had on a single occasion—those who reported having had four or more standard alcoholic drinks were categorised as heavy drinkers, those who reported between one and three drinks were coded as moderate drinkers, and those who never drank as non-users.

### Data analysis

Data analysis for this study was carried with the aid of the IBM Statistical Package for Social Sciences (SPSS) version 24.0. The analysis began with data cleaning to ensure that data entry errors were identified and removed. We then ran descriptive statistics for all categorical and continuous variables to generate frequencies and percentages. We also fitted adjusted and unadjusted logistic regression to examine the relationship between exposure to sexual violence and demographic, behavioural and family characteristics. We assess if there were multicollinearity among the variables included in our models by estimating Pearson correlation coefficient for all variables of interest, and none of them had a correlation coefficient greater than 0.5 indicating no multicollinearity. A P-value less than 0.05 was considered statistically significant, and 95% confidence intervals were estimated for odds ratios. We coded and summarised the responses to the open-ended questions on the perpetrators.

## Results

### Descriptive findings

The analysis was restricted to 451 participants who answered the question on the outcome variable. Their average age was 21.03 ± 1.61 years. Most participants were aged 20 to 24 years (79.6%), resided in the university residence (78.1%), and ever used alcohol (66.5%) (Table 1). Only a few of the participants received adequate family financial support (37.2%), had at least one child (34.6%), ever smoked cigarettes (28.2%), and ever used drugs (30.8%).

**Table 1:** Demographical and behavioural characteristics of study participants

Variables	Frequency	Percent
Age (years)		
17–19	92	20.4
20–24	359	79.6
Residence		
University residence	352	78.1
Off-campus residence	64	14.2
Come from home	35	7.8
Religiosity		
Very religious	98	21.6
Moderately religious	197	43.7
Not religious	156	21.7
Family structure		
Single parent	196	43.5
Both parents	175	38.8
Live with grandparents	49	10.9
Live with sister, uncle, brother, and aunt	31	6.9
Family financial support		
Adequate	168	37.2
Moderate	202	44.8
Insufficient	78	17.3
No support	3	0.7
Parity		
No children	295	65.4
At least one child	156	34.6
Orphanhood status		
Double orphan	48	10.6
Single orphan	147	32.6
Both parents alive	256	56.8
Living with parents		
Live with neither parents	88	19.5
Live with either parents	196	43.5
Live with both parents	167	37.0
Ever drank alcohol		
Yes	300	66.5
No	151	33.5
Currently drink alcohol		
Yes	197	43.7
No	254	56.3
Drank last week		
Yes	108	24.0
No	343	76.0
Ever smoked cigarettes or other tobacco products		
Yes	127	28.2
No	324	71.8
Currently smoke tobacco products		
Yes	51	11.3
No	400	88.7
Ever used drugs		
Yes	138	30.6
No	313	69.4
Currently use drugs		
Yes	51	11.3
No	400	88.7
Sex after alcohol		
Yes	118	26.2
No	333	73.8
Childhood exposure to sexual violence		
Yes	80	17.7
No	371	82.3

Lifetime and past-year prevalence of sexual violence were 37.9% (CI 33.4–42.5%) and 25.2% (CI 21.3–29.6%), respectively (Table 2). A higher prevalence was reported by heavy episodic drinkers of alcohol (lifetime 48.4% and past year 34.0%), those who received insufficient financial support (lifetime 58.0% and past year 35.8%) compared with non-users and those who received adequate financial support.

**Table 2:** Lifetime and past year experience of sexual violence by background characteristics

Variables	Lifetime experience of sexual violence Freq (%)	Past year experience of sexual violence Freq (%)
All	171 (37.9)	114 (25.3)
Age (yrs)		
17–19	30 (32.6)	20 (21.7)
20–24	141 (39.3)	94 (26.2)
Residence		
University residence	136 (38.6)	90 (25.6)
Off campus residence	26 (40.6)	18 (28.1)
Come from home	9 (26.7)	6 (17.1)
Family structure		
Single parent	73 (37.2)	47 (24.0)
Both parents	66 (37.7)	46 (26.3)
Live with grandparent	17 (34.7)	13 (26.5)
Live with sister and uncles	15 (48.4)	8 (25.8)
Family financial support		
Adequate	35 (20.8)	22 (13.1)
Moderate	89 (44.1)	63 (31.2)
Insufficient or no support	47 (58.0)	29 (35.8)
Parity		
One or more	70 (44.9)	49 (33.3)
No children	101 (34.2)	65 (24.4)
Ever used alcohol		
Yes	130 (43.3)	88 (29.3)
No	41 (27.2)	29 (17.2)
Ever used drug		
Yes	68 (48.3)	45 (32.6)
No	103 (32.9)	69 (22.0)
Orphanhood status		
Double orphan	23 (47.9)	13 (27.1)
Single orphan	56 (38.1)	37 (25.2)
Both parents alive	92 (35.9)	64 (25.0)
Religiosity		
Very religious	16 (16.3)	8 (8.2)
Moderately religious	78 (39.1)	52 (26.4)
Not religious	78 (50.0)	54 (34.6)
Alcohol use intensity		
Heavy drinkers	91 (48.4)	64 (34.0)
Moderate drinkers	39 (34.8)	124 (21.4)
Non-users	41 (27.2)	26 (17.2)

Freq-frequency

### Relationship between the survivors and the perpetrators

We further examined how close the perpetrators of this act were to the victims and found that two-thirds of them had some sort of relationship with the perpetrator. Many of the survivors of sexual violence (39.5%) did not mention the incidence to anyone since its occurrence (Table 3).

**Table 3:** Relationship between the victims and the perpetrators

Variables	Frequency	Percent
Who did it		
Boyfriend	37	32.2
Uncle	9	7.8
Cousin	9	7.8
Casual friend	16	13.9
Stranger	44	40.9
Told anyone about the incidence		
Yes	69	60.5
No	45	39.5

#### Multivariate findings

We used adjusted and unadjusted binary logistic regression models to examine the factors associated with exposure to sexual violence. The results of the unadjusted model, as presented in Table 4, indicate that adequate family financial support and being very religious were associated with lower odds of lifetime and past year exposure to sexual violence. However, parity, heavy episodic drinking of alcohol and drug use were significantly associated with a higher likelihood of having ever experienced sexual violence and exposure to sexual violence in the past year. In the adjusted model, adequate family financial support (AOR: 0.35; 95% CI: 0.17–0.71) and religiosity (AOR: 0.25; 95% CI: 0.11–0.57) remain protective against exposure to sexual violence. Individuals reporting adequate family financial support were 76% and 65% less likely to report lifetime and past-year exposure to sexual violence than those who received moderate or insufficient financial support. Likewise, heavy episodic drinking of alcohol remains associated with a higher likelihood of lifetime (AOR: 1.86; 95% CI: 1.07–3.25) and past year (AOR: 2.03; 95% CI: 1.10–3.75) exposure to sexual violence in the adjusted model. However, the effect of drug use and parity were no longer significant.

**Table 4:** Adjusted and unadjusted binary regression model showing predictors of lifetime and past year exposure to sexual violence

Variables	Lifetime experience of sexual violence		Past year experience of sexual violence	
	Unadjusted odds ratio	Adjusted odds ratio	Unadjusted odds	Adjusted odds
Age (yrs)				
17–19	0.75 (0.46–1.21)	1.02 (0.59–1.77)	0.78 (0.45–1.36)	1.08 (0.59–1.98)
20–24	1	1	1	1
Family financial support				
Insufficient	1	1	1	1
Moderate	0.57 (0.34–0.96)*	0.67 (0.38–1.18)	0.81 (0.47–1.40)	0.95 (0.52–1.71)
Adequate	0.19 (0.11–0.34)***	0.24 (0.13–0.46)***	0.27 (0.14–0.51)***	0.35 (0.17–0.71)*
Family structure				
Foster family	1.12 (0.66–1.91)	0.94 (0.52–1.70)	1.13 (0.62–2.05)	1.00 (0.52–1.91)
Both parents family	1.02 (0.67–1.55)	1.32 (0.75–2.32)	1.13 (0.71–1.81)	1.31 (0.70–2.45)
Single parent family	1	1	1	1
Parity				
One or more children	1.56 (1.05–2.32)*	1.27 (0.81–1.98)	1.62 (1.05–2.51)*	1.36 (0.84–2.20)
No children	1	1	1	1
Religiosity				
Very religious	0.20 (0.10–0.36)***	0.31 (0.16–0.60)*	0.17 (0.08–0.37)***	0.25 (0.11–0.57)*
Moderately religious	0.64 (0.42–98)*	0.89 (0.56–1.43)	0.68 (0.43–1.07)	0.87 (0.54–1.46)
Not Religious	1	1	1	1
Orphanhood status				
Either parent dead	1.21 (0.83–1.78)	1.07 (0.61–1.86)	1.03 (0.67–1.59)	0.92 (0.50–1.71)
Both parents alive	1	1	1	1
Drug use				
Ever used drugs	1.98 (1.32–2.98)*	1.39 (0.86–2.25)	1.71 (1.10–2.67)*	1.16 (0.69–1.94)
Never used drugs	1	1	1	1
Alcohol use intensity				
Heavy drinker	2.52 (1.59–3.98)***	1.86 (1.07–3.25)*	2.48 (1.48–4.17)*	2.03 (1.10–3.75)*
Moderate drinker	1.43 (0.84–2.43)	1.57 (0.87–2.81)	1.31 (0.71–2.43)	1.47 (0.76–2.87)
Non users	1	1	1	1

^***^P values < 0.001, *P values < 0.05

## Discussion

Sexual violence often does not receive enough attention, given that survivors hardly report the cases, even to friends or close confidants [37]. As such, relying on rape or sexual assault reporting will not provide accurate data on the prevalence of sexual violence among young people. We undertook this secondary analysis to determine the prevalence and associated factors of sexual violence among a cohort of AGYW. Our analysis showed that over a quarter of female students experience sexual violence in the past year, and approximately two out of five girls did in their lifetime. Overall, this finding highlights the high frequency at which young girls are exposed to sexual violence. A pooled analysis of the lifetime prevalence of sexual violence in South Africa academic institutions revealed a similar rate (26.2%) to our study [26]. Another study conducted in another South African Province reported a 24.9% lifetime prevalence of sexual violence among women aged 18–49 years [27]. However, the observed prevalence in this study is higher than the global prevalence of 7.2% [28], a prevalence of 17.4% in Southern Africa and other settings [38, 39]. Variations in the definition of sexual violence and the study contexts might explain the contrasting prevalence rates reported in these studies. However, it is well established that South Africa is confronted with a high prevalence of sexual violence [25, 40, 41], and our study further adds to this body of knowledge [37].

Consistent with previous studies [11, 42], our analysis shows that heavy episodic drinking was associated with an increased likelihood of exposure to sexual violence. Scholars have argued that the relationship between alcohol and sexual violence is bi-directional [43–45]. It has also been reported that male students buy alcoholic beverages for female students, pressuring them to drink above their limits and sexually abusing them when intoxicated [46]. The intoxication from alcohol could make individuals incapacitated, thereby subjecting them to rape or inappropriate and unwanted touching of genitals [8, 11]. It also renders the victims powerless and unable to identify or interpret cues, negotiate or take an active decision in the face of such incidence [47]. Also, victims of sexual violence may turn to alcohol to cope with the trauma of the incident [45]. Studies have shown that alcohol use and abuse is common among young people in South Africa [48, 49]. Thus, the high prevalence of sexual violence could somewhat be attributed to the abuse of alcohol. As such, one avenue that could be explored is stricter policies around the sales and commercialisation of alcohol and other harmful substances in and around college campuses to curb its consequential adverse effects such as sexual violence.

Notably, adequate family financial support is protective against exposure to sexual violence. The pathway through which adequate family financial support reduces the odds of exposure to sexual violence could be understood through the vulnerability lens. The primary needs of adolescents and young adults are material in nature, and money is required to meet these needs. Not getting enough money from home makes young people rate the level of support from their family as inadequate. Adequate family financial support means young people are not easily influenced by material and monetary gifts, which increases the risk of exposure to sexual violence [16, 17]. Our findings and explanation are consistent with previous studies that have established the link between poverty and sexual violence [50–52]. For example, the National Crime Victimisation Survey in the United States established that the poorest Americans are 12 times as likely as the wealthiest Americans to be exposed to sexual violence[50]. Given our finding on insufficient family financial support and exposure to sexual violence, it is paramount that any intervention being considered to tackle sexual violence must focus on addressing family and income vulnerabilities that predispose young people to sexual violence.

We also found a significant relationship between religiosity and lifetime and past year experience of sexual violence. In this study, religiosity was found to be protective against the experience of sexual violence. There are differing opinions on the impact of religion on sexual or intimate partner violence. While some scholars consider religion to be associated with positive health behaviours and, consequently, reduced sexual violence [53, 54], others have a contrary opinion. Religion, with or without cultural norms, is sometimes a guise under which victims and perpetrators hide or are hidden [55, 56]. The findings in our study could be associated with the protective effect of religion on positive health behaviours [57]. In addition, unmarried girls who frequently attend religious fellowships may embrace conservative lifestyles, including limited involvement in partying, dating and other behaviours that may increase their risks of sexual violence. Since most sexual violence occurs within intimate relationships, girls who frequently attend religious fellowship may be protected from sexual violence given their conservative lifestyle with dating [58].

Our findings on the predictors of sexual violence are consistent with the social-ecological model of violence against women [59]. The predictors of sexual violence in our study context include poverty, normative behaviour around drinking and religion, which address multiple levels of the social-ecological model. Our findings are consistent with socioecological approaches to violence prevention that bring the discourse away from blaming the individual and intervening on structural factors that contribute to sexual violence [60].

Our study established that close acquaintances were perpetrators of two-thirds of cases of sexual violence, and three-fifth of survivors did mention the incidence to someone. The fact that three-fifth of survivors mentioned it to someone underscores that many victims talk about the incidence. However, two-fifth of survivors did not mention the incidence to anyone, showing that silence around sexual violence remains a major issue. Silence exacerbates the burden of sexual violence [25]. Many victims find it difficult to report, and this has, in turn, led to less attention being paid to the matter. Several factors are responsible for this, including the fact that many of the perpetrators are close acquaintances, which makes reporting difficult [61]. Living nearby and trust in the perpetrator, despite potentially being in danger, may play a role in higher victimisation [7]. Also, the fear of stigmatisation, shame, not being believed, and the gender-inequitable societal norms as well as the difficulty of gathering sufficient evidence, further reinforce this high rate of under-reporting of sexual violence cases [30]. This challenge could be more serious in academic institutions as students could find it difficult to report cases of sexual violence due to the fear of unknown consequences, both on their academic performances and generally in terms of risk of re-traumatisation and bullying. Also, universities need to put measures in place to control such acts on college campuses and even at the hall of residences to reduce this challenge.

### Strength and limitations of the study

Data for this study were collected from university students who are more educated than the population of AGYW in the country. As such, the findings cannot be generalised across all South African AGYW. Also, given the sensitive nature of this topic, under-reporting of prevalence cannot be wholly ruled out despite providing privacy during the data collection. Lastly, the association reported in this study cannot be construed as causation, given the cross-sectional nature of the data.

## Conclusion

Our study shows that sexual violence occurs at a high frequency, requiring interventions to tackle this challenge in the study setting. Given that alcohol use and inadequate family financial support were associated with exposure to sexual violence, alcohol use reduction and conditional cash transfer programmes for indigent AGYW are potentially relevant interventions.

## Data Availability

Data will be made available by the corresponding author upon request.

## Abbreviations

AGYW: Adolescent girls and young women
STI: Sexually transmitted infections
